# Identification of single nucleotide polymorphisms of *PIK3R1* and *DUSP1* genes and their genetic associations with milk production traits in dairy cows

**DOI:** 10.1186/s40104-019-0392-z

**Published:** 2019-11-06

**Authors:** Bo Han, Yuwei Yuan, Lijun Shi, Yanhua Li, Lin Liu, Dongxiao Sun

**Affiliations:** 1 Department of Animal Genetics, Breeding and Reproduction, College of Animal Science and Technology, Key Laboratory of Animal Genetics, Breeding and Reproduction of Ministry of Agriculture and Rural Affairs, National Engineering Laboratory for Animal Breeding, China Agricultural University, No. 2 Yuanmingyuan West Road, Haidian District, Beijing, 100193 China; 2 Beijing Dairy Cattle Center, Beijing, 100192 China

**Keywords:** Chinese Holstein, *DUSP1*, Genetic association, Milk production, *PIK3R1*, SNP

## Abstract

**Background:**

Previously, phosphoinositide-3-kinase regulatory subunit 1 (*PIK3R1*) and dual specificity phosphatase 1 (*DUSP1*) were identified as promising candidate genes for milk production traits due to their being differentially expressed between the dry period and the peak of lactation in livers of dairy cows. Hence, in this study, the single nucleotide polymorphisms (SNPs) of *PIK3R1* and *DUSP1* genes were identified and their genetic associations with milk yield, fat yield, fat percentage, protein yield, and protein percentage, were investigated using 1067 Chinese Holstein cows from 40 sire families.

**Results:**

By re-sequencing the entire coding region and 2000 bp of the 5′ and 3′ flanking regions of the two genes, one SNP in the 5′ untranslated region (UTR), three in the 3′ UTR, and two in the 3′ flanking region of *PIK3R1* were identified, and one in the 5′ flanking region, one in the 3′ UTR, and two in the 3′ flanking region of *DUSP1* were found. Subsequent single-locus association analyses showed that five SNPs in *PIK3R1*, rs42590258, rs210389799, rs208819656, rs41255622, rs133655926, and rs211408208, and four SNPs in *DUSP1*, rs207593520, rs208460068, rs209154772, and rs210000760, were significantly associated with milk, fat and protein yields in the first or second lactation (*P* values ≤ 0.0001 and 0.0461). In addition, by the Haploview 4.2 software, the six and four SNPs in *PIK3R1* and *DUSP1* respectively formed one haplotype block, and the haplotype-based association analyses showed significant associations between their haplotype combinations and the milk traits in both two lactations (*P* values ≤ 0.0001 and 0.0364). One SNP, rs207593520(T/G), was predicted to alter the transcription factor binding sites (TFBSs) in the 5′ flanking region of *DUSP1*. Further, the dual-luciferase assay showed that the transcription activity of allele T in rs207593520 was significantly higher than that of allele G, suggesting the activation of transcriptional activity of *DUSP1* gene by allele T of rs207593520. Thus, the rs207593520 SNP was highlighted as a potential causal mutation that should be further verified.

**Conclusions:**

We demonstrated novel and significant genetic effects of the *PIK3R1* and *DUSP1* genes on milk production traits in dairy cows, and our findings provide information for use in dairy cattle breeding.

## Background

Genomic selection has been widely applied in dairy cattle breeding. The evaluation system with DNA marker technology and genomics has increased the rate of genetic progress for economic traits [1]. Zhang et al. illustrated that the available quantitative trait locus (QTL) lists detected by hundreds of genome-wide association studies (GWASs) and QTL mapping studies improved the performance of genomic prediction in dairy cattle [2]. To date, a large number of QTLs and genetic associations have been reported for milk traits (http://www.animalgenome.org/cgi-bin/QTLdb/index). Nowadays, RNA sequencing (RNA-Seq) has been proved to be an effective tool to identify crucial functional genes for complex traits in humans, domestic animals, and plants [3–5]. In a previous study, the liver transcriptomes of Chinese Holstein cows in the dry period, early lactation, and peak of lactation, were analyzed and the expression of phosphoinositide-3-kinase regulatory subunit 1 (*PIK3R1*; *P* value = 0.0009) and dual specificity phosphatase 1 (*DUSP1*; *P* value = 0.00005) were found significantly decreased and increased in the peak of lactation compared to that in the dry period, respectively. Moreover, the *PIK3R1* gene was linked with metabolic gene ontology (GO) terms and pathways, including protein phosphatase binding, protein transport, positive regulation of glucose import, and AMPK (adenosine 5′-monophosphate (AMP)-activated protein kinase), insulin, PI3K-Akt [phosphatidylinositol 3′-kinase (PI3K)-Akt], mTOR [mammalian (mechanistic) target of rapamycin], and Jak-STAT (janus kinase/signal transducers and activators of transcription) signaling pathways, and the *DUSP1* gene was linked with inactivation of mitogen-activated protein kinase (MAPK) activity, protein binding, protein dephosphorylation, and MAPK signaling pathway [6], implying that the two genes were involved in milk metabolisms.

*PIK3R1* is the regulatory subunit 1 of PI3K that plays an important role in the metabolic actions of insulin in the PI3K signaling pathway. *DUSP1*, which dephosphorylates c-Jun N-terminal kinase and p38 MAPK, is a negative regulator of MAPK involved with lipid, glucose and energy metabolisms, mitochondrial biogenesis, immune, and various diseases [7–12]. In addition, the *PIK3R1* gene is located on chr.20:14.0466 cM within a distance of 1.95 ~ 3.97 cM to the reported QTLs that have large effects on protein yield [13, 14], and it is 0.38 ~ 2.80 Mb away from five SNPs significantly associated with milk production traits, ARS-BFGL-NGS-16696, BTA-51542-no-rs, ARS-BFGL-NGS-45127, ARS-BFGL-NGS-29910, and Hapmap50995-BTA-51556, identified by the previous GWAS [15]. The *DUSP1* gene (chr.20: 5.47596 cM) was found to be within the reported QTL regions that were confirmed to have large genetic effects on fat yield [14] and protein yield [13], and within 0.044 ~ 3.50 Mb from the four SNPs, ARS-BFGL-NGS-48030, Hapmap54098-rs29010434, Hapmap49207-BTA-51446, and Hapmap36217-SCAFFOLD290026_21689, significantly associated with milk traits [15]. These data suggest that *PIK3R1* and *DUSP1* might be potential candidate genes for milk production traits in dairy cows. Hence, in this study, the single nucleotide polymorphisms (SNPs) of the two genes were identified by re-sequencing and then their genetic effects on milk yield, fat yield, fat percentage, protein yield, and protein percentage were investigated using single-locus and haplotype-based association analyses.

## Materials and methods

### Animal, sample and phenotypic data collection

Chinese Holstein cows were maintained with the same feeding conditions in 22 dairy farms belonging to the Sanyuan Lvhe Dairy Farming Centre (Beijing, China), and 1067 cows from 40 sire families were selected for the study. The semen of 40 sires and blood samples of 1067 cows respectively were collected for SNP discovery and association analysis. DNAs were extracted from the semen and blood samples using a salt-out procedure and TIANamp Blood DNA Kits (Tiangen, Beijing, China), respectively. The quantity and quality of the extracted DNA samples were measured by using a NanoDrop 2000 spectrophotometer (Thermo Scientific, Hudson, NH, USA) and gel electrophoresis respectively. The phenotypic data for 305-day milk yield, fat yield, fat percentage, protein yield, and protein percentage were provided by the Beijing Dairy Cattle Centre (http://www.bdcc.com.cn/; Beijing, China). The descriptive statistics of the phenotypic values for milk production traits in the first and second lactations are presented in Table 1.

**Table 1:** Descriptive statistics of the phenotypic values for milk production traits

	Lactation	Milk yield, kg	Fat yield, kg	Fat percentage, %	Protein yield, kg	Protein percentage, %
Mean	1	10,379.81	350.55	3.387	313.78	3.024
	2	10,859.85	390.12	3.599	322.20	2.970
Standard deviation	1	1476.18	60.70	0.423	48.20	0.198
	2	1876.61	83.85	0.504	57.63	0.192
Maximum	1	14,505.68	537.97	4.742	457.53	3.508
	2	16,512.08	658.25	5.431	467.03	3.538
Minimum	1	6057.96	184.80	2.059	157.73	2.257
	2	4756.94	145.05	2.212	137.86	2.199
Coefficient of variation	1	14.22	17.31	12.490	15.36	6.548
	2	17.28	21.49	14.004	17.89	6.465

### SNP identification and genotyping

The Primer3 (http://bioinfo.ut.ee/primer3-0.4.0/) was used with the bovine reference genome sequence (NC_037347.1) template to design primers that would amplify the entire coding region and 2000 bp of the 5′ and 3′ flanking regions of the *PIK3R1* and *DUSP1* genes (Additional file 1). Primers were synthesized by the Beijing Genomics Institute (BGI, Beijing, China). Forty semen DNAs with equal concentrations (2.5 ng) for each DNA, were randomly mixed into two pools of 20 sires each and used for all the polymerase chain reactions (PCR). PCR was performed with the conditions presented in Additional file 1. After the amplification, the purified PCR products were sequenced using an ABI3730XL DNA analyser (Applied Biosystems, Foster City, CA, USA), and the sequencing data were analyzed by using CHROMAS (version 2.23) to identify the potential SNPs. The identified SNPs were then individually genotyped using Sequenom MassArray for all the 1067 cows by matrix-assisted laser desorption/ionization time of flight mass spectrometry (MALDI-TOF MS, Sequenom MassARRAY, Bioyong Technologies Inc., HK).

### Linkage disequilibrium (LD) estimation and association analyses

The extent of LD between the identified SNPs were estimated using Haploview 4.2 (Broad Institute of MIT and Harvard, Cambridge, MA, USA). The single-locus and haplotype-based association analyses in the first and second lactations for milk production traits were performed using the mixed procedure of SAS 9.13 software with the following animal model: *y* = *μ* + *HYS* + *b* × *M* + *G* + *a* + *e*, where, *y* is the phenotypic value of each trait for each cow; *μ* is the overall mean; *HYS* is the fixed effect of farm, year and season of calving; *b* is the regression coefficient of covariant *M*; *M* is the fixed effect of calving month; *G* is the genotype or haplotype combination effect; *a* is the individual random additive genetic effect, distributed as $\mathrm{N}\ \left(0,\mathbf{A}{\updelta}_a^2\right)$, with the additive genetic variance ${\updelta}_a^2$; and *e* is the random residual, distributed as $\mathrm{N}\ \left(0,\mathbf{I}{\updelta}_{\mathrm{e}}^2\right)$, with identity matrix I and residual error variance ${\updelta}_{\mathrm{e}}^2$. Each trait was analyzed separately and each SNP/haplotype block was also fitted separately. Bonferroni correction was applied, and the significant level was equal to the raw *P* value divided by number of genotypes or haplotype combinations. Furthermore, the additive (*a*), dominant (*d*), and substitution (*α*) effects were calculated using the following formulas: $\mathrm{a}=\frac{\mathrm{AA}-\mathrm{BB}}{2};\mathrm{d}=\mathrm{AB}-\frac{\mathrm{AA}+\mathrm{BB}}{2};\upalpha =\mathrm{a}+\mathrm{d}\ \left(\mathrm{q}-\mathrm{p}\right)$, where, *AA*, *BB*, and *AB* are the least square means of the milk production traits in the corresponding genotypes, *p* is the frequency of allele A, and *q* is the frequency of allele B [16].

### Transcription factor binding site (TFBS) prediction and dual-luciferase assay

The MatInspector (https://david.ncifcrf.gov/home.jsp) was used to predict the changes of the TFBSs (Matrix similarity threshold, MST > 0.90) caused by the SNPs in the 5′ flanking or UTR region of the two genes. The fragments containing the binding sites of transcription factor in the 5′ flanking region of the *DUSP1* gene were synthesized by Genewiz company (Suzhou, China) and cloned into the pGL4.14 Luciferase Assay Vector (Promega, Madison, WI). The plasmid constructs were sequenced to confirm the integrity of each insertion, and purified with an Endo-free Plasmid Maxi Kit (ComWin Biotech, Beijing, China). The human embryonic kidney 293 T (HEK 293 T) cells were grown in Dulbecco’s modified Eagle’s medium (DMEM; Gibco, Life Technologies, Carlsbad, CA) supplemented with 10% fetal bovine serum (FBS; Gibco), and maintained at 37 °C in a humidified incubator with 5% CO_2_. Cells were seeded in the 24-well plates at approximately 2 × 10^5^ cells per well before transfection. For each well, 500 ng of the constructed plasmid was co-transfected along with 10 ng of pRL-TK Renilla luciferase reporter vector (Promega) using Lipofectamine 3000 (Invitrogen, CA, USA) according to the manufacturer’s protocol. All the experiments were performed in triplicate. The cells were harvested at 48 h after transfection and the activities of firefly and renilla luciferases were measured using a Dual-Luciferase Reporter Assay System (Promega) on a Modulus microplate multimode reader (Turner Biosystems, CA, USA). The normalized luciferase data (firefly/renilla) were used to calculate the average statistics of the replicates.

## Results

### Identification of polymorphisms

The entire coding region and 2000 bp of 5′ and 3′ flanking regions of the *PIK3R1* and *DUSP1* genes were re-sequenced. Six SNPs were identified in *PIK3R1*, including rs42590258 in the 5′ untranslated region (UTR), rs210389799, rs208819656, and rs41255622 in the 3′ UTR, and rs133655926 and rs211408208 in the 3′ flanking region. Four SNPs were found in *DUSP1*, including rs207593520 in the 5′ flanking region, rs208460068 in the 3′ UTR, and rs209154772 and rs210000760 in the 3′ flanking region (Table 2). The number of cows with different genotypes in the first and second lactations are presented in Table 2, and the allelic and genotypic frequencies are also shown.

**Table 2:** Detailed information about the identified SNPs

Gene	SNP	GenBank No.	Position (UMD3.1)	Location	Allele	Allelic frequency	Genotype	Genotypic frequency
*PIK3R1*	c.*208G > A	rs42590258	chr20:11410254	5’UTR	G	0.64	AA	0.13
					A	0.36	AG	0.46
							GG	0.41
	c.*2776 T > C	rs210389799	chr20:11329079	3´UTR	T	0.73	CC	0.07
					C	0.27	TC	0.41
							TT	0.52
	c.*2962 T > C	rs208819656	chr20:11328893	3´UTR	T	0.72	CC	0.07
					C	0.28	CT	0.42
							TT	0.51
	c.*6275 T > A	rs41255622	chr20:11325580	3´UTR	T	0.52	AA	0.23
					A	0.48	AT	0.50
							TT	0.27
	g.11323546C > T	rs133655926	chr20:11323546	3´flanking region	C	0.52	CC	0.27
					T	0.48	TC	0.51
							TT	0.22
	g.11323118G > A	rs211408208	chr20:11323118	3´flanking region	G	0.72	AA	0.07
					A	0.28	AG	0.42
							GG	0.51
*DUSP1*	g.4453141 T > G	rs207593520	chr20:4453141	5´flanking region	G	0.35	GG	0.13
					T	0.65	GT	0.44
							TT	0.43
	c.*1505G > A	rs208460068	chr20:4449625	3´UTR	A	0.31	AA	0.07
					G	0.69	AG	0.47
							GG	0.46
	g.4448024C > T	rs209154772	chr20:4448024	3´flanking region	C	0.68	CC	0.46
					T	0.32	TC	0.45
							TT	0.09
	g.4447105C > G	rs210000760	chr20:4447105	3´flanking region	C	0.68	CC	0.46
					G	0.32	CG	0.45
							GG	0.09

Note: *UTR* untranslated region

### Single-locus association analyses with five milk production traits

For *PIK3R1* gene, the single-locus association results (Table 3) showed that the rs42590258 was significantly associated with milk yield (*P* values = 0.0373 and 0.0233) and fat yield (*P* values = 0.0461 and 0.0065) in the first and second lactations, and protein percentage in the first lactation (*P* value = 0.0131), respectively. The SNP, rs208819656, was strongly associated with protein yield in the both lactations (*P* values = 0.0258 and 0.0117), fat yield in the first lactation (*P* value = 0.0272), and milk yield in the second lactation (*P* value = 0.0207), respectively. The rs41255622 and rs133655926 were significantly associated with milk, fat and protein yields in the first lactation (*P* values: 0.0032 ~ 0.0437). The rs211408208 had a strong association with fat yield in the first lactation (*P* value = 0.0111). While, the rs210389799 had no significant association with the five milk traits (*P* values > 0.05). Interestingly, there were no association between the six SNPs of *PIK3R1* and the fat and protein percentage traits, except rs42590258 was associated with the protein percentage in the first lactation (*P* value = 0.0131). The allele additive, dominant, and substitution effects of the six SNPs of *PIK3R1* gene were also calculated, and their significant associations with milk, fat and protein yields were found (*P* values < 0.05; Additional file 2).

**Table 3:** Associations of the SNPs in *PIK3R1* and *DUSP1* genes with milk production traits in two lactations in Chinese Holstein (LSM ± SE)

Gene	SNP	Lactation	Genotype(*n*)	Milk yield, kg	Fat yield, kg	Fat percentage, %	Protein yield, kg	Protein percentage, %
*PIK3R1*	rs42590258	1	AA (135)	10,462 ± 86.13^a^	349.88 ± 3.66^a^	3.35 ± 0.04	306.8 ± 2.67	2.93 ± 0.01^Aa^
			AG (473)	10,266 ± 65.34^b^	343.3 ± 2.9^ab^	3.36 ± 0.03	303.29 ± 2.11	2.96 ± 0.01^ab^
			GG (428)	10,314 ± 65.48^ab^	342.19 ± 2.91^b^	3.33 ± 0.03	305.32 ± 2.12	2.96 ± 0.01^Bb^
			*P* value	0.0373	0.0461	0.3398	0.1916	0.0131
		2	AA (99)	10,959 ± 98.39^a^	391.1 ± 4.16^ab^	3.59 ± 0.04	324.99 ± 3.03	2.97 ± 0.01
			AG (328)	10,751 ± 68.47^ab^	391.5 ± 3.02^Aa^	3.65 ± 0.03	318.46 ± 2.2	2.97 ± 0.01
			GG (297)	10,695 ± 69.91^b^	383.63 ± 3.08^Bb^	3.6 ± 0.03	318.94 ± 2.24	2.99 ± 0.01
			*P* value	0.0233	0.0065	0.0538	0.0541	0.0979
	rs210389799	1	CC (70)	10,283 ± 104.72	338.75 ± 4.38	3.31 ± 0.04	301.96 ± 3.19	2.94 ± 0.01
			TC (427)	10,362 ± 67.57	342.2 ± 2.99	3.31 ± 0.03	305.69 ± 2.18	2.95 ± 0.01
			TT (548)	10,381 ± 63.46	345.82 ± 2.83	3.34 ± 0.03	306.91 ± 2.06	2.96 ± 0.01
			*P* value	0.5914	0.0705	0.2506	0.1953	0.4421
		2	CC (55)	10,619 ± 119.13	380.41 ± 4.96	3.62 ± 0.05	314 ± 3.62	2.96 ± 0.02
			TC (287)	10,770 ± 72.29	385.05 ± 3.18	3.59 ± 0.03	320.02 ± 2.32	2.98 ± 0.01
			TT (384)	10,685 ± 65.96	384.05 ± 2.94	3.62 ± 0.03	317.03 ± 2.14	2.98 ± 0.01
			*P* value	0.2598	0.6072	0.6018	0.1141	0.5929
	rs208819656	1	CC (72)	10,185 ± 102.59	337.79 ± 4.42^ab^	3.34 ± 0.04	297.83 ± 3.25^Aa^	2.96 ± 0.01
			CT (419)	10,298 ± 66.79	339.69 ± 2.99^a^	3.32 ± 0.03	303.08 ± 2.19^ab^	2.95 ± 0.01
			TT (516)	10,332 ± 63.92	344.74 ± 2.87^b^	3.35 ± 0.03	305.28 ± 2.09^Bb^	2.96 ± 0.01
			*P* value	0.2816	0.0272	0.2915	0.0258	0.3219
		2	CC (58)	10,537 ± 117.02^a^	389.48 ± 3.43	3.62 ± 0.05	312.93 ± 3.57^a^	2.97 ± 0.02
			CT (286)	10,822 ± 72.57^b^	391.17 ± 3.04	3.61 ± 0.03	321.96 ± 2.32^b^	2.98 ± 0.01
			TT (376)	10,694 ± 67.37^ab^	387.81 ± 3.35	3.62 ± 0.03	317.97 ± 2.19^ab^	2.98 ± 0.01
			*P* value	0.0207	0.5115	0.7832	0.0117	0.8131
	rs41255622	1	AA (231)	10,265 ± 72.87^a^	338.83 ± 3.17^a^	3.32 ± 0.03	302.24 ± 2.31^Aa^	2.95 ± 0.01
			AT (508)	10,309 ± 64.3^a^	342.69 ± 2.86^ab^	3.34 ± 0.03	304.52 ± 2.08^a^	2.96 ± 0.01
			TT (270)	10,449 ± 70.93^b^	346.79 ± 3.11^b^	3.33 ± 0.03	308.75 ± 2.26^Bb^	2.96 ± 0.01
			*P value*	0.013	0.0135	0.7073	0.0032	0.7936
		2	AA (174)	10,845 ± 76.65	384.11 ± 3.33	3.63 ± 0.03	320.8 ± 2.5	2.98 ± 0.01
			AT (354)	10,737 ± 69.06	386.59 ± 3.06	3.64 ± 0.03	319.91 ± 2.21	2.98 ± 0.01
			TT (196)	10,670 ± 78.59	381.95 ± 3.4	3.6 ± 0.03	320.73 ± 2.44	2.97 ± 0.01
			*P* value	0.1056	0.2695	0.428	0.8883	0.5442
	rs133655926	1	CC (275)	10,451 ± 70.63^a^	347.43 ± 3.1^a^	3.34 ± 0.03	307.9 ± 2.25^a^	2.95 ± 0.01
			TC (519)	10,313 ± 64.02^b^	343.66 ± 2.85^ab^	3.35 ± 0.03	304.46 ± 2.08^ab^	2.96 ± 0.01
			TT (229)	10,295 ± 73.94^ab^	340.67 ± 3.22^b^	3.33 ± 0.03	303.06 ± 2.34^b^	2.95 ± 0.01
			*P* value	0.0267	0.0437	0.6163	0.0358	0.6543
		2	CC (200)	10,845 ± 76.65	384.11 ± 3.33	3.57 ± 0.03	320.18 ± 2.42	2.96 ± 0.01
			TC (358)	10,737 ± 69.06	386.59 ± 3.06	3.62 ± 0.03	318.32 ± 2.23	2.97 ± 0.01
			TT (173)	10,670 ± 78.59	381.95 ± 3.4	3.6 ± 0.03	316.28 ± 2.48	2.97 ± 0.01
			*P value*	0.1056	0.2695	0.2678	0.2898	0.6173
	rs211408208	1	AA (69)	10,135 ± 105.87	339.36 ± 4.59^ab^	3.37 ± 0.04	297.57 ± 3.24	2.95 ± 0.01
			AG (419)	10,252 ± 67.09	342.8 ± 3.04^a^	3.34 ± 0.03	301.17 ± 2.16	2.95 ± 0.01
			GG (508)	10,260 ± 63.18	348.14 ± 2.86^b^	3.37 ± 0.03	302.97 ± 2.05	2.96 ± 0.01
			*P* value	0.4168	0.0111	0.2459	0.1178	0.1653
		2	AA (57)	10,713 ± 117	385.34 ± 4.88	3.61 ± 0.05	317.64 ± 3.56	2.96 ± 0.02
			AG (286)	10,859 ± 72.14	389.01 ± 3.16	3.59 ± 0.03	322.05 ± 2.3	2.97 ± 0.01
			GG (371)	10,743 ± 66.59	386.73 ± 2.96	3.62 ± 0.03	317.79 ± 2.16	2.97 ± 0.01
			*P* value	0.1481	0.5837	0.6063	0.0645	0.871
*DUSP1*	rs207593520	1	GG (128)	10,310 ± 87.86	337.1 ± 3.75^a^	3.27 ± 0.04	304.66 ± 2.73	2.95 ± 0.01^ab^
			GT (444)	10,372 ± 65.07	344.51 ± 2.89^b^	3.33 ± 0.03	305.05 ± 2.1	2.94 ± 0.01^a^
			TT (431)	10,397 ± 65.53	345.64 ± 2.91^b^	3.34 ± 0.03	307.76 ± 2.12	2.96 ± 0.01^b^
			*P* value	0.5322	0.0201	0.1115	0.1487	0.026
		2	GG (99)	11,256 ± 98.44^A^	395.73 ± 4.18^Aa^	3.65 ± 0.04	323.58 ± 3.05^a^	2.97 ± 0.01
			GT (303)	10,964 ± 69.52^Bb^	384.24 ± 3.08^Bb^	3.59 ± 0.03	318.36 ± 2.24^ab^	2.97 ± 0.01
			TT (310)	10,833 ± 71.81^Bb^	385.59 ± 3.14^b^	3.61 ± 0.03	316.61 ± 2.29^b^	2.97 ± 0.01
			*P* value	<.0001	0.0073	0.2932	0.0418	0.7827
	rs208460068	1	AA (76)	10,603 ± 105.06^a^	347.69 ± 4.41	3.29 ± 0.04	313.22 ± 3.21^a^	2.96 ± 0.01
			AG (473)	10,354 ± 65.04^b^	344.92 ± 2.88	3.35 ± 0.03	305 ± 2.1^Bb^	2.95 ± 0.01
			GG (467)	10,369 ± 64.51^b^	343.71 ± 2.86	3.33 ± 0.03	306.61 ± 2.08^ab^	2.96 ± 0.01
			*P* value	0.0338	0.5601	0.2866	0.0132	0.2481
		2	AA (59)	11,344 ± 118.56^A^	399.21 ± 4.95^Aa^	3.54 ± 0.05	336.32 ± 3.61^A^	2.97 ± 0.02
			AG (332)	10,842 ± 68.11^B^	392.67 ± 3.01^Aa^	3.62 ± 0.03	321.62 ± 2.19^B^	2.97 ± 0.01
			GG (339)	10,617 ± 69.61^C^	382.41 ± 3.08^B^	3.62 ± 0.03	315.07 ± 2.24^C^	2.97 ± 0.01
			*P* value	<.0001	<.0001	0.2052	<.0001	0.969
	rs209154772	1	CC (466)	10,307 ± 64.33^a^	341.63 ± 2.86	3.33 ± 0.03	305.42 ± 2.08^a^	2.96 ± 0.01
			TC (458)	10,316 ± 64.98^a^	344.11 ± 2.88	3.35 ± 0.03	304.36 ± 2.1^Aa^	2.95 ± 0.01
			TT (91)	10,552 ± 98.79^b^	345.62 ± 4.16	3.29 ± 0.04	312.43 ± 3.03^Bb^	2.96 ± 0.01
			*P* value	0.0203	0.3403	0.1808	0.0089	0.1257
		2	CC (339)	10,639 ± 69.67^Aa^	384.61 ± 3.08^Aa^	3.63 ± 0.03	316.76 ± 2.24^Aa^	2.98 ± 0.01
			TC (318)	10,794 ± 68.91^Ab^	391.15 ± 3.04^c^	3.63 ± 0.03	320.77 ± 2.22^Aa^	2.98 ± 0.01
			TT (70)	11,295 ± 111.38^B^	401.97 ± 4.67^Bb^	3.58 ± 0.05	334.82 ± 3.41^B^	2.97 ± 0.02
			*P* value	<.0001	0.0001	0.4368	<.0001	0.6954
	rs210000760	1	CC (462)	10,343 ± 64.92^a^	345.15 ± 2.89	3.35 ± 0.03	306.37 ± 2.1^a^	2.96 ± 0.01
			CG (458)	10,319 ± 64.75^Aa^	346.4 ± 2.87	3.37 ± 0.03	304.42 ± 2.09^Aa^	2.95 ± 0.01
			GG (91)	10,585 ± 98.43^Bb^	348.72 ± 4.15	3.3 ± 0.04	312.86 ± 3.02^Bb^	2.95 ± 0.01
			*P* value	0.0118	0.5743	0.1534	0.0052	0.1864
		2	CC (340)	10,614 ± 69.01^A^	385.45 ± 3.05^Aa^	3.64 ± 0.03	316.05 ± 2.22^Aa^	2.98 ± 0.01
			CG (319)	10,826 ± 68.53^B^	392.49 ± 3.03^b^	3.63 ± 0.03	321.5 ± 2.2^Ab^	2.98 ± 0.01
			GG (71)	11,302 ± 109.96^C^	401.5 ± 4.61^Bb^	3.57 ± 0.04	335.23 ± 3.36^B^	2.97 ± 0.02
			*P* value	<.0001	0.0003	0.2737	<.0001	0.8597

Note: The number in the bracket represents the number of cows for the corresponding genotype; *P* value shows the significance for the genetic effects of SNPs; ^a, b, c^ within the same column with different superscripts means *P* value < 0.05; ^A, B, C^ within the same column with different superscripts means *P* value < 0.01

As for the *DUSP1* gene, the genetic associations between the four identified SNPs and five milk production traits were analyzed. The results showed that the four SNPs were mainly significantly associated with milk, fat and protein yields (*P* values ≤ 0.0001 and 0.0418), and only one SNP, rs207593520, had strongly association with protein percentage in the first lactation (*P* value = 0.026). In the first lactation, rs208460068, rs209154772, and rs210000760 were significantly associated with milk and protein yields (*P* values: 0.0052 ~ 0.0338), while, rs207593520 had associations with fat yield (*P* value = 0.0201) and protein percentage (*P* value = 0.026). In the second lactation, the four SNPs, rs207593520, rs208460068, rs209154772, and rs210000760, were strongly associated with milk, fat and protein yields (*P* values ≤ 0.0001 and 0.0418; Table 3). Further, the additive, dominant and substitution effects of the four SNPs were analyzed, and their significant associations with milk, fat and protein yields were found (*P* values < 0.05; Additional file 2).

### Haplotype-based association analyses with five milk traits

**Fig. 1:**
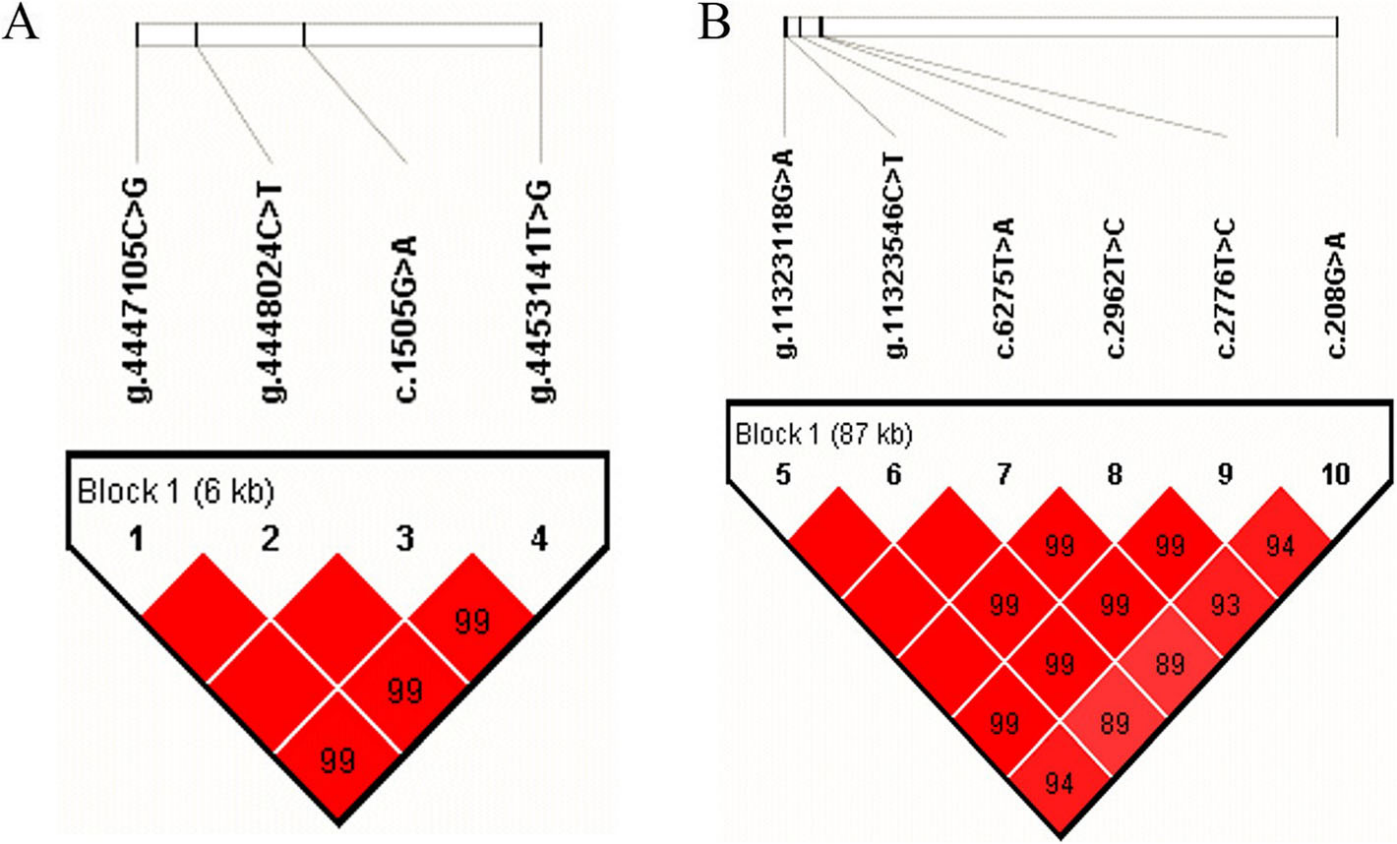
Linkage disequilibrium estimated among the SNPs in *DUSP1* (**a**; D′ =0.99 ~ 1.00) and *PIK3R1* (**b**; D′ =0.89 ~ 1.00). The blocks indicate haplotype blocks and the text above the horizontal numbers is the SNP names. The values in boxes are pairwise SNP correlations (D′), while bright red boxes without numbers indicate complete LD (D′ = 1)

The extent of LD between the six identified SNPs in *PIK3R1* were estimated using Haploview 4.2, and six SNPs were found highly linked (D′ > 0.89; Fig. 1) in one block. The haplotype block was formed by five haplotypes, H1 (GCTTTA), H2 (GTATTG), H3 (ATACCG), H4 (GCTTTG), and H5 (GTATTA), with the frequency of 34.7%, 18.7%, 26.8%, 17.6%, and 1.3%, respectively. The haplotype-based association analysis showed that the haplotype block was significantly associated with milk yield (*P* value < 0.0001), fat yield (*P* value = 0.0002), protein yield (*P* value < 0.0001), and protein percentage (*P* value = 0.0002) in the first lactation, and milk yield (*P* value = 0.0006), fat percentage (*P* value = 0.0305), protein yield (*P* value = 0.003), and protein percentage (*P* value = 0.0142) in the second lactation, respectively (Table 4).

**Table 4:** Associations of haplotype blocks with milk production traits in two lactations in Chinese Holstein (LSM ± SE)

Block	Lactation	Haplotype combination (*n*)	Milk yield, kg	Fat yield, kg	Fat percentage, %	Protein yield, kg	Protein percentage, %
*PIK3R1*	1	H1H1 (130)	10,360 ± 89.61^bc^	343.34 ± 3.81^ab^	3.33 ± 0.04	304.19 ± 2.78^abcd^	2.94 ± 0.01^Aa^
		H1H2 (144)	10,048 ± 84.93^Aa^	337.91 ± 3.63^a^	3.38 ± 0.03	298.02 ± 2.64^Aa^	2.97 ± 0.01^abc^
		H1H3 (192)	10,187 ± 79.12^ac^	336.9 ± 3.41^Aa^	3.33 ± 0.03	300.14 ± 2.49^ACa^	2.95 ± 0.01^Aac^
		H1H4 (130)	10,474 ± 86.18^Bb^	350.09 ± 3.68^Bb^	3.36 ± 0.04	312.38 ± 2.68^Bb^	2.99 ± 0.01^bc^
		H2H3 (124)	10,238 ± 87.58^abc^	335.93 ± 3.73^Aa^	3.3 ± 0.04	301.63 ± 2.72^ACDacd^	2.95 ± 0.01^ac^
		H2H4 (65)	10,327 ± 109.41^abc^	346.65 ± 4.57^ab^	3.37 ± 0.04	310.8 ± 3.33^BCbd^	3.01 ± 0.02^Bb^
		H3H3 (68)	10,150 ± 107.13^abc^	335.16 ± 4.49^a^	3.32 ± 0.04	299.42 ± 3.27^ACDad^	2.95 ± 0.02^abc^
		H3H4 (111)	10,448 ± 93.43^Bbc^	347.85 ± 3.97^ab^	3.35 ± 0.04	310.36 ± 2.89^BDbc^	2.98 ± 0.01^abc^
		*P* value	<.0001	0.0002	0.4417	<.0001	0.0002
	2	H1H1 (96)	11,118 ± 100.05^Aa^	390.74 ± 4.23	3.53 ± 0.04^a^	328.3 ± 3.08^Aa^	2.96 ± 0.01^ab^
		H1H2 (106)	10,643 ± 95.67^Bb^	392.23 ± 4.06	3.69 ± 0.04^b^	315.26 ± 2.96^Bb^	2.97 ± 0.01^ab^
		H1H3 (126)	11,056 ± 91.29^Aa^	395.21 ± 3.9	3.58 ± 0.04^ab^	324.96 ± 2.84^ab^	2.95 ± 0.01^a^
		H1H4 (86)	10,863 ± 100.55^ab^	395.32 ± 4.26	3.64 ± 0.04^ab^	322.5 ± 3.11^ab^	2.98 ± 0.01^ab^
		H2H3 (89)	10,920 ± 98.93^ab^	393.52 ± 4.19	3.6 ± 0.04^ab^	325.88 ± 3.05^b^	2.99 ± 0.01^ab^
		H2H4 (42)	10,915 ± 133.11^ab^	385.35 ± 5.5	3.52 ± 0.05^ab^	328.33 ± 4.01^ab^	3.01 ± 0.02^b^
		H3H3 (52)	10,841 ± 124.39^ab^	388.26 ± 5.19	3.6 ± 0.05^ab^	321.81 ± 3.78^ab^	2.97 ± 0.02^ab^
		H3H4 (73)	10,726 ± 110.54^ab^	387.3 ± 4.64	3.6 ± 0.04^ab^	319.54 ± 3.38^ab^	2.99 ± 0.02^ab^
		*P* value	0.0006	0.4862	0.0305	0.003	0.0142
*DUSP1*	1	H1H1 (136)	10,202 ± 87.64^ab^	333.67 ± 3.74	3.29 ± 0.04	301.78 ± 2.72^ab^	2.96 ± 0.01^ab^
		H1H2 (240)	10,309 ± 73.31^ab^	343.11 ± 3.19	3.35 ± 0.03	303.68 ± 2.32^ab^	2.95 ± 0.01^ab^
		H1H3 (241)	10,235 ± 74.22^ab^	339.15 ± 3.22	3.34 ± 0.03	300.48 ± 2.34^Aa^	2.94 ± 0.01^Aa^
		H2H2 (112)	10,261 ± 90.23^ab^	338.92 ± 3.83	3.33 ± 0.04	305.83 ± 2.79^ab^	2.99 ± 0.01^Bb^
		H2H3 (238)	10,203 ± 73.37^a^	341.72 ± 3.19	3.38 ± 0.03	301.47 ± 2.33^a^	2.96 ± 0.01^ab^
		H3H3 (79)	10,526 ± 104.21^b^	344.29 ± 4.37	3.29 ± 0.04	311.35 ± 3.18^Bb^	2.96 ± 0.01^ab^
		*P* value	0.0364	0.0678	0.109	0.0054	0.0131
	2	H1H1 (101)	11,043 ± 98.24^ADa^	402.02 ± 4.16^Aa^	3.66 ± 0.04	329.32 ± 3.03^ACa^	2.97 ± 0.01
		H1H2 (165)	10,632 ± 82.2^Bb^	384.09 ± 3.55^BCb^	3.62 ± 0.03	316.74 ± 2.59^BDd^	2.97 ± 0.01
		H1H3 (156)	10,995 ± 82.56^Aa^	398.32 ± 3.55^Aa^	3.62 ± 0.03	327.79 ± 2.58^Aa^	2.98 ± 0.01
		H2H2 (77)	10,248 ± 106.51^Cc^	376.09 ± 4.48^Bb^	3.65 ± 0.04	306.23 ± 3.26^Bb^	2.98 ± 0.01
		H2H3 (167)	10,798 ± 82.69^ab^	395.31 ± 3.57^ACa^	3.66 ± 0.03	321.26 ± 2.6^ADad^	2.97 ± 0.01
		H3H3 (59)	11,485 ± 118.31^Dd^	408.46 ± 4.93^Aa^	3.57 ± 0.05	341.32 ± 3.6^Cc^	2.97 ± 0.02
		*P* value	<.0001	<.0001	0.4432	<.0001	0.9643

Note: H means haplotype; the number in the bracket represents the number of cows for the corresponding haplotype combination; *PIK3R1*: H1 (GCTTTA), H2 (GTATTG)), H3 (ATACCG), H4 (GCTTTG), and H5 (GTATTA); *DUSP1*: H1 (CCGG), H2 (CCGT), and H3 (GTAT); *P* value shows the significance for genetic effects among the haplotype blocks; ^a, b, c, d^ within the same column with different superscripts means *P* value < 0.05; ^A, B, C, D^ within the same column with different superscripts means *P* value < 0.01

As for *DUSP1*, the four SNPs were found highly linked in one haplotype block with D´ > 0.99 (Fig. 1). The haplotype block was consist of three haplotypes, H1 (CCGG), H2 (CCGT), and H3 (GTAT), with the frequency of 35.5%, 32.9%, and 30.6%, respectively. The haplotype block was significantly associated with milk yield (*P* value = 0.0364), protein yield (*P* value = 0.0054) and percentage (*P* value = 0.0131) in the first lactation, and milk, fat and protein yields (*P* values < 0.0001) in the second lactation, respectively. While, no genetic association was observed between the haplotype block and fat percentage in the both two lactations (*P* values > 0.1; Table 4).

### Transcriptional activity of *DUSP1* increased by allele T of rs207593520

**Fig. 2:**
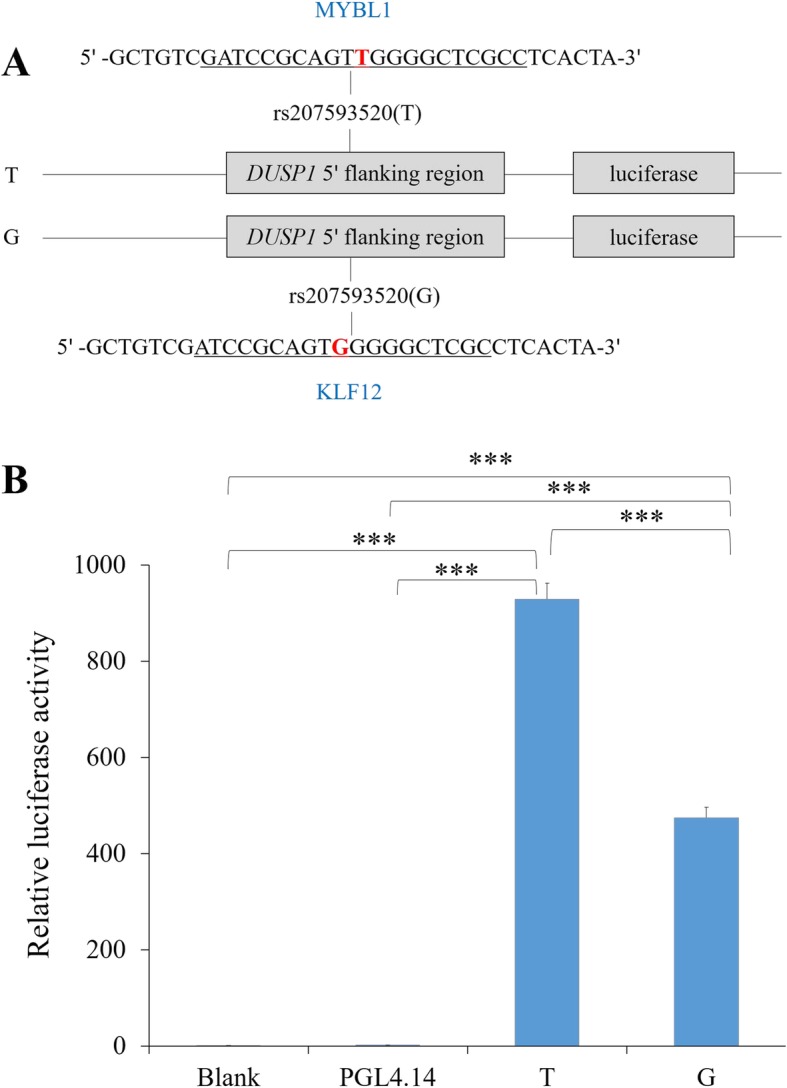
Dual-luciferase activity assay. **a** Sketches of recombinant plasmids with rs207593520 (T/G) in the 5′ flanking region of *DUSP1* gene. Underlined nucleotides represent the transcription factor binding site sequences of MYBL1 or KLF12 (in blue), and the nucleotide in red was the SNP. **b** Luciferase activity analysis of the recombinant plasmids in HEK 293 T cells. PGL4.14 was the empty vector, Blank was the blank cell, and *** *P* value < 0.0001

The MatInspector was used to predict the changes of TFBSs due to the SNPs in the 5′ flanking or UTR region of the two genes, and the allele T of rs207593520 in *DUSP1* was found to create the binding sites for the transcription factor MYB proto-oncogene like 1 (MYBL1; MST = 0.91), and the allele G invented the binding sites for kruppel like factor 12 (KLF12; MST = 0.96; Fig. 2a). Further, two plasmids containing allele T or G in the rs207593520 were synthesized for dual-luciferase assay to observe the changes of the transcriptional activity of *DUSP1*. As the results shown in Fig. 2b, the luciferase activities of two constructs were significantly higher than that of the empty vector (PGL4.14) and blank control (*P* value < 0.0001), confirming the regulatory role of rs207593520. Moreover, the relative luciferase activity of allele T was significantly higher than that of allele G (*P* value < 0.0001), implying that the allele T of rs207593520 in *DUSP1* might have higher transcriptional activity than the allele G (Fig. 2b).

## Discussion

Our previous RNA-Seq work considered *PIK3R1* and *DUSP1* as the candidate genes for milk production traits, and this follow-up investigation first demonstrated that the polymorphisms of the two genes were both mainly significantly associated with milk, fat and protein yields. To our knowledge, the *PIK3R1* occupies a center role in the insulin signaling pathway, and it was reported to be associated with alterations in glucose and insulin homeostasis [17]. It can recruit protein kinase A to the lipid droplet in conveying endogenous glucocorticoid-induced lipolysis [18]. Studies also revealed that *PIK3R1* influences the serum leptin and body fat [19], apolipoprotein B, and low density lipoprotein cholesterol [20] in female. *DUSP1* is belongs to the MKP phosphatase family, and it regulates the MAPK signaling pathway by the dephosphorylation. Studies have uncovered an important regulatory role for *DUSP1* in hepatic lipid metabolism [7, 8, 10]. Mice lacking *DUSP1* were resistant to the acquisition of a fatty liver suggesting that *DUSP1* negatively regulates hepatic fatty acid oxidation [10]. Lawan et al. [9, 21] have reported the contribution of *DUSP1* for glucose and energy metabolisms. These data collectively illustrate that *PIK3R1* and *DUSP1* participate in substance metabolisms, especially lipid metabolisms, and their polymorphisms were found to be significantly associated with milk, fat and protein yields by the association analyses in this study. Additionally, the haplotype analyses were generally used to the genetic variation studies [22, 23]. Our haplotype-based association analyses showed that the six and four SNPs of *PIK3R1* and *DUSP1* were respectively highly linked, and the haplotype block of the two genes were all significantly associated with milk traits in Holstein cows, which were consistent with the genetic associations of SNPs with the milk traits.

In the present study, the allele T of rs207593520 in the 5′ flanking region of *DUSP1* was predicted to invent the TFBSs for MYBL1 and the allele C for KLF12, and further, the transcriptional activity of *DUSP1* was found significantly increased by the allele T of rs207593520 in the dual-luciferase assay. It is generally known that the SNPs in TFBSs could lead to allele-specific binding of transcription factors thereby activating or suppressing the gene expression [24–26]. MYB proteins are nuclear DNA-binding proteins that act as transcriptional transactivators of many genes [27], and MYBL1 has been reported as a master regulator of meiotic genes that are involved in multiple meiotic processes [28]. The transcription factor MYBL1 activates the murine tissue-specific lactate dehydrogenase expression by binding the cAMP-responsive element site [29]. In addition, MYBL1 can act as a transcriptional repressor to suppress the expression of multiple anthocyanin pigment pathway genes [30]. Transcription factor KLF12 is a member of KLFs family, which regulates gene transcription through binding to the CACCC sequence of target genes [31, 32]. Studies also showed that it can bind to the promoter regions of target genes and represses their expression [32–35]. KLF12 acts to negatively regulate the expression of the decidual marker genes decidual prolactin and insulin like growth factor binding protein 1 in human endometrial stromal cells [32]. KLF12 binds to the promoter region of leukemia inhibitory factor and directly represses its transcription [35]. While, KLF12 can directly activate the expression of early growth response protein 1 to promote the colorectal cancer growth [36]. These data suggest that the transcription factors MYBL1 and KLF12 can activate or repress the expression of their target genes, however, MYBL1 mainly acts as an activator, and KLF12 prefers to be a repressor. Based on our results, we speculated that MYBL1 might activate the expression of *DUSP1* by binding the TFBSs caused by allele T of rs207593520, thereby regulating the milk production, and KLF12 might inhibit the *DUSP1* expression through binding the TFBSs caused by the allele G to affect the milk production. Hence, the SNP, rs207593520, may be a potential causal mutation for milk yield traits in dairy cows because of its ability to change the transcriptional activity of *DUSP1*, and the further functional studies are required to validate its role.

Genomic selection is widely used in dairy cattle breeding, and the development of efficient SNP markers can improve the accuracy of the selection. Studies have shown that the SNPs in the functional genes significantly influenced the milk production traits in dairy cattle [37–40]. Thus, the SNPs with large genetic effects on milk traits could be used as markers to increase the selection efficiency in specific dairy cattle populations. Certainly, the significant SNPs of *PIK3R1* and *DUSP1* genes identified in this study could also be used for genomic selection in dairy cattle.

## Conclusion

In conclusion, this is the first study to reveal the significant genetic effects of *PIK3R1* and *DUSP1* genes on milk production traits in dairy cows, and the valuable SNPs could be used for the genomic selection in dairy cattle. In addition, the rs207593520 in *DUSP1* was highlighted as a functional mutation for milk traits that could change the transcriptional activity of *DUSP1* gene. Further, the functional validation experiments should be performed to reveal the molecular regulatory mechanisms of *PIK3R1* and *DUSP1* genes on milk synthesis metabolism.

## Supplementary information

**Additional file 1. Table S1.** Primers and procedures for PCR used in SNP identification.

**Additional file 2. Table S2.** Additive, dominant and allele substitution effects of SNPs on milk production traits in Chinese Holstein.

## Data Availability

All relevant data are available within the article and its additional files.

## Abbreviations

a: additive
AMPK: Adenosine 5′-monophosphate (AMP)-activated protein kinase
d: dominant
DMEM: Dulbecco’s modified Eagle’s medium
DUSP1: Dual specificity phosphatase 1
FBS: Fetal bovine serum
GO: Gene Ontology
GWAS: Genome-wide association studie
Jak-STAT: Janus kinase/signal transducers and activators of transcription
KLF12: Kruppel like factor 12
LD: LINKAGE disequilibrium
MALDI-TOF MS: matrix-assisted laser desorption/ionization time of flight mass spectrometry
MAPK: Mitogen-activated protein kinase
mTOR: mammalian (mechanistic) target of rapamycin
MYBL1: MYB proto-oncogene like 1
PCR: Polymerase chain reaction
PI3K-Akt: Phosphatidylinositol 3′-kinase (PI3K)-Akt
PIK3R1: Phosphoinositide-3-kinase regulatory subunit 1
QTL: Quantitative trait locus
RNA-Seq: RNA sequencing
SNPs: Single nucleotide polymorphisms
TFBSs: Transcription factor binding sites
UTR: Untranslated region
α: substitution
